# Identifying and understanding the health and social care needs of older adults with multiple chronic conditions and their caregivers: a scoping review

**DOI:** 10.1186/s12877-018-0925-x

**Published:** 2018-10-01

**Authors:** Katherine S. McGilton, Shirin Vellani, Lily Yeung, Jawad Chishtie, Elana Commisso, Jenny Ploeg, Melissa K. Andrew, Ana Patricia Ayala, Mikaela Gray, Debra Morgan, Amanda Froehlich Chow, Edna Parrott, Doug Stephens, Lori Hale, Margaret Keatings, Jennifer Walker, Walter P. Wodchis, Veronique Dubé, Janet McElhaney, Martine Puts

**Affiliations:** 1 Lawrence S. Bloomberg Faculty of Nursing, University of Toronto, Toronto, ON Canada; 2 Toronto Rehabilitation Institute, University Health Network, 550 University Avenue, Toronto, ON M6K 2R7 416 597 3422 (2500) Canada; 3 Institute of Health Policy, Management and Evaluation, University of Toronto, Toronto, ON Canada; 4 School of Nursing, McMaster University, Hamilton, ON Canada; 5 Division of Geriatric Medicine, Dalhousie University, Halifax, NS Canada; 6 Gerstein Information Science Centre, University of Toronto, Toronto, ON Canada; 7 Canadian Centre for Health and Safety in Agriculture, University of Saskatchewan, Saskatoon, SK Canada; 8 The Change Foundation, Toronto, ON Canada; 9 Laurentian University, Sudbury, ON Canada; 10 Institute for Better Health, Trillium Health Partners, Mississauga, ON Canada; 11 Faculty of Nursing, Université de Montréal, Montreal, Quebec, Canada; 12 Health Sciences North Research Institute and Northern Ontario School of Medicine, Sudbury, ON Canada; 13 Rehabilitation Sciences Institute, University of Toronto, Toronto, ON Canada

**Keywords:** Scoping review, Multimorbidity, Older adults, Caregivers, Health care providers, Needs

## Abstract

**Background:**

As the population is aging, the number of persons living with multiple chronic conditions (MCC) is expected to increase. This review seeks to answer two research questions from the perspectives of older adults with MCC, their caregivers and their health care providers (HCPs): 1) What are the health and social care needs of community-dwelling older adults with MCC and their caregivers? and 2) How do social and structural determinants of health impact these health and social care needs?

**Methods:**

We conducted a scoping review guided by a refinement of the Arksey & O’Malley framework. Articles were included if participants were 55 years or older and have at least two chronic conditions. We searched 7 electronic databases. The data were summarized using thematic analysis.

**Results:**

Thirty-six studies were included in this review: 28 studies included participants with MCC; 12 studies included HCPs; 5 studies included caregivers. The quality of the studies ranged from moderate to good. Five main areas of needs were identified: need for information; coordination of services and supports; preventive, maintenance and restorative strategies; training for older adults, caregivers and HCPs to help manage the older adults’ complex conditions; and the need for person-centred approaches. Structural and social determinants of health such as socioeconomic status, education and access influenced the needs of older adults with MCC.

**Conclusion:**

The review highlights that most of the needs of older adults with MCC focus on lack of access to information and coordination of care. The main structural and social determinants that influenced older adults’ needs were their level of education/health literacy and their socioeconomic status.

**Electronic supplementary material:**

The online version of this article (10.1186/s12877-018-0925-x) contains supplementary material, which is available to authorized users.

## Background

Many older adults live with multiple chronic conditions (MCC), also known as multimorbidity [1, 2]. While there are several definitions of multimorbidity, it is defined in this review as the presence of two or more chronic medical conditions which may negatively impact an individual’s daily living, particularly with higher numbers of coexisting conditions [3]. Older adults living with MCC often rely on the support of informal caregivers to help them manage their daily lives [4, 5]. Caregiving for older adults, without the appropriate supports, can negatively affect an individual’s financial, emotional and psychological wellbeing [6].

Factors related to social and structural determinants of health can further worsen the challenges of managing complex health issues for older adults with MCC [7]. This is especially true for older women, ethno cultural minorities, Indigenous persons, persons with cognitive impairment (CI), persons with lower socioeconomic status (SES), or persons living in rural or remote communities [8, 9]. Currently, not enough is known about the needs of older adults with MCC and their caregivers and how different determinants of health influence their needs as most conceptualizations of multimorbidity focus on the biomedical dimensions of MCC [10].

To date, there are syntheses of existing evidence on the spectrum of multimorbidity and implications for care [11]; occurrence, causes and consequences of multimorbidity [12]; tools to assess patient treatment priorities [13]; interventions to improve outcomes for persons with MCC [14]; and a review of chronic care models to reorganize care for patients with MCC [15]. However, to our knowledge, there is no review on the health and social care needs of community-dwelling older adults with MCC and their caregivers. Therefore, a comprehensive review is needed to inform the development of interventions designed to meet the needs, and hence promote the quality of life, of older adults with MCC and their caregivers.

In light of this gap, we undertook a scoping review to summarize the available research studies on the health and social care needs of community-dwelling older adults with MCC and their caregivers. The review seeks to answer two research questions from the perspectives of older adults with MCC, their caregivers and their health care providers (HCPs):

1. What are the health and social care needs of community-dwelling older adults with MCC and their caregivers?
2. How do social and structural determinants of health – such as gender, socioeconomic status, or level of education – impact the health and social care needs?

The scoping review methodology was chosen because it was 1) flexible in that it allowed for the inclusion of qualitative and quantitative studies [16]; 2) unrestrictive, thus allowing for the exploration of widely varied topics such as patient and caregivers needs, as well as determinants of health [16]; 3) a systematic method for summarizing and identifying gaps in existing literature [16]; and 4) citizen engaged because it includes a stakeholder consultation to inform research and subsequently evidence informed interventions [17].

## Methods

The protocol for our scoping review has been published [18] but is briefly summarized below. The scoping review methods framework outlined by Arksey and O’Malley [16] and refined by Levac et al. [17], Colquhoun et al. [19] and Daudt et al. [20] was used. The framework includes six steps: 1) identifying the research questions (listed above); 2) identifying relevant literature; 3) study selection; 4) charting the data; 5) collating, summarizing and reporting the results; 6) consulting with key stakeholders and translating knowledge. Below we briefly summarize each step.

To identify relevant literature, two academic health sciences librarians (APA and MG) prepared the search strategy in consultation with the research team. The databases searched include OVID Medline (1946 to 2017, including Epub Ahead of Print, and In Process & Other Non-Indexed Citations), OVID Embase (1947 to 2017), OVID PsycINFO (1806 to 2017), OVID Social Work Abstracts (1968 to 2017), EBSCO CINAHL Plus with Full Text (1981 to 2017), EBSCO AgeLine (1966 to 2017), and Cochrane Central. The search strategies were translated using each database platform’s command language, controlled vocabulary, and appropriate search fields. MeSH terms, EMTREE terms, APA thesauri terms, CINAHL headings, and text words were used for the search concepts of health and social care needs and priorities, Indigenous populations and multimorbidity.

We applied a modified adult age filter to the Medline strategy [21]. This filter was translated and applied to the other databases. The filters were not validated. Language limits were applied to capture English, French, Dutch, and German articles; and the final searches were completed in May 2017. For the full Medline strategy, see Additional file 1: Table S1. Additionally, we searched the reference lists of included studies. Covidence systematic review software was used to facilitate the review (www.covidence.org).

In terms of preparing the co-authors to assist with the review, the two lead PIs (KM and MP) and the librarian held training sessions with the researchers and stakeholders to ensure they understood definitions of health and social care and were familiar with the inclusion and exclusion criteria and how to use Covidence. During this phase we went through titles and abstracts together (*n* = 20) to ensure they were prepared. Conflicts were decided upon by the two lead PIs, KM and MP. The definition of social care that guided this review was from the WHO world report on Ageing and Health: Social care address the needs associated with performance of the activities of daily living, connection to one’s social networks such as family, friends and community; access to social programs for supports in poverty, unemployment, old age and disability to optimize social protection [7].

**Fig. 1:**
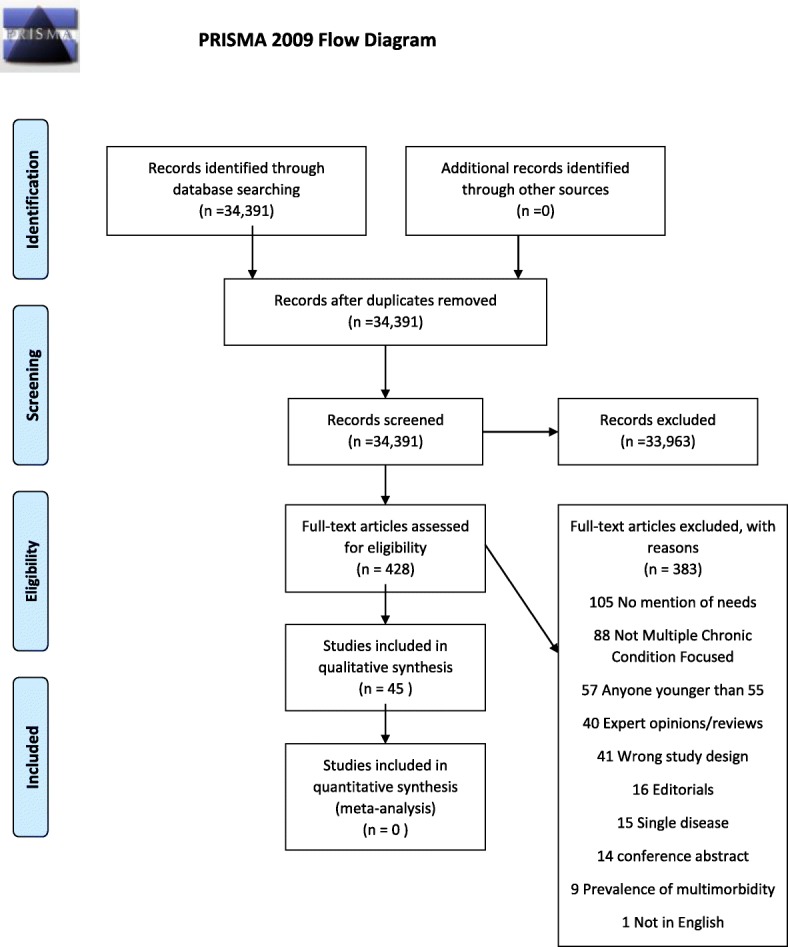
PRISMA 2009 Flow Diagram

Studies were selected through a two-step process using the selection criteria below. First, each of the titles and corresponding abstracts were independently reviewed by two team members. Then, two reviewers independently assessed the relevant full text articles (see Fig. 1 PRISMA flow chart). In cases of disagreement between reviewers, one of the two principal investigators (MP and KM) resolved the conflict.

The inclusion criteria included:

- Studies which reported on health and/or social care needs of older adults living with MCC or on health and/or social care needs of caregivers of older adults living with MCC and/or identified needs/areas for improvement.
- Any type of primary study (quantitative, qualitative or mixed methods); involving community dwelling older adults aged > 55 years; studies that included a wider age range, but the mean/median age was > 55 years; studies which included a sub-group analysis for this population.

In light of the fact that Indigenous persons experience multiple and complex health conditions at younger ages than other populations, we included all literature that focused on persons 55 years of age or older living with MCC, in order to capture relevant literature related to the care needs of ageing Indigenous persons [22].

The exclusion criteria included:

- Expert opinions, editorials, and materials that did not include original data.
- Published in other languages than English, French, Dutch and German

A data abstraction form using Microsoft Excel software was created to guide data extraction. Furthermore, codes representing the World Health Organization’s (WHO) definition of structural and social determinants of health [23] were included to map the studies that identified these determinants as important considerations when identifying needs and preferences of older adults with MCC. The data abstraction form was pilot tested and refined by the researchers (KM, MP, JC) to ensure consistency. Two reviewers independently abstracted the relevant information from the studies, with one researcher confirming the information.

During the testing of the data abstraction process it became clear that the studies focused on needs and preferences of Indigenous older adults and how data were collected were conceptually distinct from those studies focusing on groups of non-Indigenous older adults. Thus, considering these differences, it was decided to separate the review results into two papers and results concerning the needs and preferences of Indigenous older adults with MCC will be summarized in a separate paper. Team members who have experience in Indigenous health research took the lead on the other paper (JW, AC).

Data extracted included: details on the study (type of study, aim of study, origin, response rate etc.), study characteristics (study setting), patient and/or caregiver characteristics (age, gender, ethnicity, location, number and types chronic conditions), involvement of caregiver, health and social care needs, and (if categorized) categories/themes used. Since there was substantial heterogeneity among the included studies, the data were summarized using thematic analysis [24]. Using an iterative process, the first author developed descriptive codes, which were grouped together into a smaller number of categories to draw conclusions. The codes were discussed with the entire team until we reached consensus. Once the codes were agreed upon, the codes and their smaller number of categories were shared with a small group of expert qualitative researchers (JP, VD) and they were revised to minimize bias. This process was repeated to find patterns across the three different sub-groups, older adults, caregivers and HCPs. The quality of the research studies was assessed using the Mixed Methods Appraisal Tool (MMAT) [25]. The MMAT allows inclusion of qualitative, quantitative and mixed methods studies with quality criteria relevant to each study design. As we aimed to provide a comprehensive overview of needs, no study was excluded based on the MMAT score alone.

Finally, in accordance with Step 6 of our scoping review framework, we organized a stakeholder consultation meeting on 22 May 2017. We included older adults with MCC (*n* = 3), caregivers (*n* = 3), HCPs (*n* = 3), representatives of provincial organisations (*n* = 3), and primary care organizations (*n* = 2) to provide feedback on the findings and to offer suggestions for next steps. For this meeting we presented the results and asked the stakeholders specific questions related to:1) their thoughts, reflection and opinions on the information presented; 2) if there were any unmet health and social care needs, priorities and preferences of older adults with multiple chronic conditions living in the community and their caregivers, that was not highlighted in our presentation; 3) and additional social determinants of health (such as income, social support networks; education; employment/working conditions; gender; and culture) that may have impacted the health and social care needs of older persons with multiple chronic conditions; 4) and their insights about future research ideas to explore in this topic area. Two groups were developed so more sharing could occur and equal representation from both persons living with MCC and their caregivers and HCPs. The groups were facilitated by the two PIs. Notes were taken by two team members, compared, synthesized and then content analyzed. Their comments were summarized and are presented below. Additionally, to ensure we had stakeholder engagement throughout the project, three of the stakeholders who attended this meeting were selected prior to the funding of the research project. In fact one older adult with MCC and one caregiver provided testimonials during the application phase on the importance of this research. They also assisted with reviewing of abstract titles as we provided training and support for them.

## Results

The perspectives of older adults, their caregivers, and their HCPs, were considered when identifying needs of older adults with MCC. The following sections highlight the characteristics of the studies, the details of the study participants across all the studies that were reviewed and the thematic analysis of the findings.

### Characteristics of included studies

In this review 34,391 abstracts were retrieved and reviewed by two independent reviewers (see Table 1). 428 articles were selected for full text review and 45 were retained. Of these 9 were focused on Indigenous older adults so for this review, a total of 36 articles were included. Thirty studies were qualitative studies [26–55], four were cross-sectional studies [56–59], one was mixed method study [60] and one was a secondary qualitative data analysis [61] (Table 1). Twenty two studies were conducted in North America [26, 28–30, 33, 35, 37, 38, 41, 43, 46–52, 55, 57–60], ten in Europe [31, 32, 34, 39, 40, 44, 45, 54, 56, 61] and three in Australia/New Zealand [27, 42, 53].

**Table 1:** Characteristics of included studies

Author and year	Continent	Inclusion criteria	Study design	Data collection methods	Sampling Strategy	Analysis methods used
Adeniji 2015	Europe	Recruited from 4 large general practices in UK. Identified from registers of long term conditions, have at least two MCC (of COPD, coronary heart disease, diabetes, osteoarthritis, and depression)	Cross Sectional Observational	Mailed questionnaires	Convenience	Descriptive statistics and multivariable regression analysis
Ancker 2015	North American	Adult English speaking patients with MCC, as well as health care providers with experience providing care for patients with MCC	Qualitative	One to one Interviews	Purposive	Grounded theory, thematic analysis
Ansari 2014	Australia	One or more pre-existing comorbidity along with a new diagnosis of COPD in last 24 months; age 40–85; history of smoking; from primary care setting	Qualitative	One to one interview	Purposive	Thematic analysis
Bardach 2012	North America	Physicians from family medicine and internal medicine specialties were recruited from rural and urban practices, community and academic settings 1 obstetrics-gynecology physician was included, as they serve as primary care provider for some women.	Qualitative	One to one, semi structured interviews	Purposive	Content, Thematic analysis
Barstow 2015	North America	OT were identified by those attending an online forum and at a national conference who provided direct care to older adults with low vision > 1 year. Older adults with confirmed low vision from an age-related eye disease, aged 65 years and over, with at least 1 comorbid condition and no more than mild cognitive impairment	Mixed Method (cross sectional observation and qualitative)	Online surveys for OTs; one to one interviews with older adults	Convenience for OTs; Purposive for older adults	Descriptive statistics for surveys. Content analysis for qualitative.
Bayliss 2003	North America	Individuals were recruited through flyers in family medicine practices in Denver for participants who self-identified as having 2 or more chronic illnesses. They screened out those with active terminal illness, HIV, and uncontrolled psychiatric illnesses.	Qualitative	One to one interviews	Purposive	Qualitative comparative analysis
Bayliss 2007	North America	Participants of a health maintenance organization who were 65 years or older and had a diagnosis of diabetes, depression and osteoarthritis for a period of 2 years prior to the study and they were drawn from disease specific registries validated against ICD codes	Cross sectional	Survey	Convenience for survey; random for qualitative interview.	Descriptive statistics, Multivariate linear regression
Beverly 2011	North America	Mentally alert community-dwelling adults, aged 60 years or older, reporting a diagnosis of Type 2 diabetes and the presence of one or more chronic conditions in addition to diabetes	Qualitative	Eight 90 min Focus groups of 2–6 patients	Purposive	Thematic analysis
Bunn 2017	Europe	They recruited purposive samples of people living with dementia and at least one of the following three conditions: diabetes, stroke or vision impairment. They also recruited family carers and healthcare professionals who organise and deliver care for people with stroke, diabetes and VI in primary and secondary care.	Qualitative	Focus groups with HCPs; one to one interviews with patients and caregivers; one to one interviews with HCPs as well.	Purposive	Thematic and ontent analysis informed by theories of continuity of care and access to care.
Burton 2016	Europe	Eligible patients were identified from clinics and support groups but no inclusion criteria reported	Qualitative	One to one interviews	Not clear.	Thematic analysis
Cheraghi-Sohi 2013	Europe	Patients who had osteoarthritis (OA) whose transcript contained narrative of one or more condition in addition to OA and include information pertaining to condition prioritization.	Secondary analysis of qualitative data	Secondary data of one to one qualitative interviews	Purposive	Amplified secondary analysis, content analysis
Clarke 2014	North America	Aged 70 years and older and had at least 3 chronic conditions of which one of them had to be arthritis/ back problems/ cataracts/ glaucoma/ diabetes/ heart disease	Qualitative	One to one interviews	Purposive	Thematic analysis (Marshall and Rossman’s (2006) seven key analytic procedure)
Coventry 2014	Europe	To include 5 patients per criterion: age, gender, combination of illnesses and level of deprivation. Socioeconomic deprivation (defined by Index of Multiple Deprivation), number and type of long term conditions, age and gender. HCP inclusion criteria: Tried to recruit 5 in each criterion: deprivation status of the practice area; role (i.e. salaried family physician, practice nurse); and number of years’ experience.	Qualitative	One to one interviews	Convenience- HCP. Purposive- patients	Thematic analysis
DiNapoli 2016	North America	Aged 50 years and over with at least a CIRS-G 2 score in three or more organ systems and MMSE> 24 and no deficit in language skills, bipolar disorder or other chronic psychotic disorders or no other neurodegenerative disorders	Qualitative	One to one interview	Purposive	Descriptive statistics, thematic analysis
Fortin 2005	North America	Adult patients without cognitive impairment or uncontrolled illnesses, have at least 4 chronic conditions and not followed by other researchers.	Qualitative	Focus groups	Purposive	Other
Fried 2008	North America	Aged 65 and older and were taking five or more medications daily; undergoing treatment for multiple conditions; English speaking. People with severe hearing loss or cognitive impairment, defined as inability to remember two or more items on a three-item test of short-term recall were excluded	Qualitative	Focus groups	Purposive	Thematic and content analyses using constant comparative method
Gill 2014	North America	Patients: 65 years or older, diagnosed with 2 or more chronic conditions, with an informal caregiver who participated in the patient’s healthcare; spoke English as a first language; could provide consent	Qualitative	One to one interviews	Purposive	Inductive thematic analysis with saturation of themes
Grundberg 2016	Europe	Being a district nurse with experience with caring for community-dwelling homebound older adults with MCC	Qualitative	One to one interviews, focus groups	Snowballing	Content analysis
Hansen 2015	Europe	Community dwelling; 3 or more coexisting chronic conditions; being a regular patient of the participating family physician practice; ability to participate in interview (no blindness/ deafness); ability to speak German; no lethal illness in last 3 months; ability to consent e.g. no dementia; no participation in other studies at the current time;	Qualitative	Focus groups	Purposive	Content analysis
Kuluski 2013	North America	65 years or older; ability to communicate in English; two or more chronic diagnoses; ability to give informed consent; an informal caregiver who agreed to participate in an interview	Qualitative	One to one interview	Purposive	Descriptive statistics; Thematic analysis
Lo 2016	Australia	Patients with diabetes and chronic kidney disease (stages 3–5, eGFR < 60 mL/min/1.73 m2) and their carers; capable of giving consent and stable mental state. These patients from Monash health, Alfred health in Melbourne and the royal north shore and concord hospital in Sydney.	Qualitative	Focus groups for patients; semi structured interviews for carers	Purposive	Generic inductive thematic approach
Loeb 2003	North America	Mentally alert community-dwelling adults, aged 55 or older, who reported the presence of at least two chronic conditions	Qualitative	Focus Groups	Purposive	Thematic and content analyses
Mason 2016	Europe	Having advanced multimorbidity defined as having multiple life-limiting illnesses or progressively deteriorating health due to several long-term conditions. Patients with moderate to severe cognitive impairment were excluded. Patients were asked to nominate a family carer who consented separately	Qualitative	One to one interviews. Serial interviews at 8–12 week intervals. Among 87 interviews, 42 with patients alone, 2 with carers alone, 43 were joint interviews	Purposive	Constructivist thematic analysis.
McDonnall 2016	North America	Recruited from a previous study, from the centre for Deaf-Blind youths and adults, and ads and electronic discussion groups. 55 years and older who have dual sensory loss	Cross sectional	Survey	Purposive	Descriptive statistics Open-ended responses were independently coded by two the authors, and discrepancies were discussed until agreement was reached
Morales-Asencio 2016	Europe	Patients experiencing situations with high probability of complexity, such as the coexistence of several chronic diseases impacting quality of life, the frequent interaction with health services, or the existence of health/social determinants. Gender was also included as a selection criterion because of the proven differences in significance granted by men and women to their health care events and to their process experience	Qualitative	One to one interview with caregiver present	Purposive	Qualitative inductive content analysis
Naganathan 2016	North America	65 years of age or older, and diagnosed with two or more chronic conditions, patient capacity to provide informed consent, presence of informal care-giver and patient English proficiency.	Qualitative	One to one interview	Convenience	Descriptive statistics, thematic analysis
Noël 2005	North America	8 primary care clinics in 4 regions in the US were selected. The study sites were chosen based on known regional variations in veteran’s health and differences in clinic size and organization. Four clinics were in large metropolitan settings and four were in rural areas. 4/8 were based in tertiary care hospitals and the others were free standing community clinics. Patients were invited by primary care physician if they had 2 or more diseases, have no severe cognitive/mental health illnesses.	Qualitative	Focus groups	Purposive	Thematic analysis
Ravenscroft 2010	North America	Recruitment criteria: (1) adults (19 years or older) with diagnosed stage 1 to 4 Chronic Kidney Disease (CKD), (2) attending a clinic for management of their CKD, (3) coexisting diabetes mellitus and/or Cardiovascular disease, or both, and (4) capable of communicating in English	Qualitative	One to one interviews	Purposive	Thematic analysis
Richardson 2016	North America	Be at least 18 years of age or older, (2) have a diabetes diagnosis, and (3) have at least two other diagnosed chronic conditions. Excluded patients with cognitive deficits, uncontrolled psychiatric illness.	Qualitative	One to one interview, chart review	Purposive	Descriptive statistics, content analysis with naturalistic approach
Roberge 2016	North America	Clinicians from 3 different university affiliated family health teams in Quebec. Clinicians: 1) provision of services to patients with chronic diseases; 2) at least 12 months of clinical experience; Patients: 1) age 18 years or older, 2) presence of a chronic disease (e.g. diabetes, arthritis, chronic obstructive pulmonary disease); 3) depression or anxiety disorder (panic disorder, agoraphobia, social anxiety disorder or generalized anxiety disorder) in the past 2 years according to clinician’s diagnosis; 4) good knowledge of French or English; 5) having a family physician in one of the three clinics. Exclusion criteria for patients were the inability to provide consent, cognitive impairment, and a history of manic episodes or a psychotic disorder.	Qualitative	One to one interview	Purposive	Thematic analysis
Roberto 2005	North America	Women 65 years or older with two or more of heart disease, diabetes or osteoporosis.	Qualitative	One to one interview	Purposive	Thematic analysis- based on life course perspective and trajectory model of chronic illness
Ryan 2016	North America	Those who have high needs (combinations of major chronic conditions, under 65 and disabled, frail elderly with multiple functional limitations; insurance status).	Cross sectional observational	One to one interviews	Random-The 2016 Commonwealth Fund Survey of High-Need Patients was conducted by SSRS from June 22 to September 14, 2016, as a part of SSRS’s weekly, nationally representative omnibus survey	Prevalence reported only
Schoenberg 2011	North America	41 and over; diagnosis of two or more chronic illnesses, have ‘just enough money to get by’ or ‘not enough money to make ends meet’.	Qualitative	One to one interview	Purposive	Thematic and content analyses
Sheridan 2012	New Zealand	Based on ethnicity (Maori, Pacific, Asian, or New Zealand European), 50 years or older, two or more chronic conditions, admitted to hospital two or more times for five or more bed days between Jan and Dec 2008	Qualitative	One to one interviews	Purposive	Qualitative Descriptive approach
Smith 2010	Europe	Family Physicians who also trained medical trainees were selected from Trinity College Dublin; Pharmacists were selected from pharmacists attending a chronic disease management resource group	Qualitative	Focus groups	Purposive	Thematic analysis
Zulman 2015	North America	Individuals who receive care at an academic medical center or at a Veterans Affair facility in Northern California. eligibility criteria for the focus groups (≥3 chronic conditions and experience using technology to help them care for their health or manage their health care) Did not exclude based on age, health status, functional/cognitive status.	Qualitative	Focus groups	Purposive	Thematic and Content analyses

*CIRS-G* Cumulative Illness Rating-Geriatrics

*COPD* Chronic obstructive pulmonary disease

*eGFR* estimated Glomerular Filtration Rate

*HCP* Health care provider

*MCC* Multiple Chronic Conditions

*MMSE* Mini Mental State Examination

*OT* Occupational therapist

*SD* Standard deviation

We sought information from older adults with MCC, their caregivers and health care providers about needs of older adults with MCC and some researchers included more than one perspective in their studies [26, 31, 34, 38, 40–42, 44, 46, 50, 53, 60]. Thirty-four studies included participants with MCC [26, 27, 29–38, 40–53, 55–61] and the sample size ranged from 8 [60] to 1274 participants [58] with a total of 3058 participants in the studies. Seven studies included caregivers of older adults with MCC [31, 38, 41, 42, 44–46] and the sample sizes varied from 8 [42] to 33 [31] with a total of 137 participants. In the twelve studies that included health care providers [26, 28, 31, 34, 38–41, 46, 50, 54, 60], the sample sizes ranged from 4 [38, 41] to 59 [60] and a total of 201 HCPs participated. Included papers were published between 2003 and 2017.

### Characteristics of the study participants

The mean age of older adults ranged from 55 to 64 in four studies [26, 35, 52, 55], 65 to 74 in nine studies [27, 34, 36, 42, 43, 45, 56, 57, 61], and 75 to 84 in twelve studies [30–33, 37, 38, 40, 41, 44, 46, 51, 60] (see Table 2). Seven studies reported age ranges [29, 47–50, 53, 59]. Mean age and age ranges of older adults with MCC are presented in Table 1. The percentage of female older adults with MCC ranged from 6% [49] to 100% [51]. Of the 36 studies included in the review, 22 studies included at least 50% females as their study participants [26, 28–30, 32, 33, 36, 37, 39, 43, 45, 48, 50–53, 56–61]. Two studies did not report the proportion of female respondents [34, 54].

**Table 2:** Characteristics of study participants

Author and year	Sample size and mean age (age range)	% Female	Diseases	Number of diseases	Ethnicity	SES	Living situation/Marital status	Education	Other
Older adult characteristics									
Adeniji 2015	*N* = 486. mean 70 (range 31–91)	52	COPD, coronary heart disease, diabetes, osteoarthritis, depression	Median: 7; range: 2–20	Not reported	13% in paid jobs; no further details. 68% owned cars	Not reported	60% completed school/GCSE as a minimum level of education	
Ancker 2015	*N* = 22 mean age = 64 (range 37–89)	50	Hypertension, heart disease, chronic pain, depression, asthma, HIV, hepatitis C & diabetes	Mean = 3.5 (SD 1.5). Minimum 2 conditions.	32% black	32% had Medicare, 32% had Medicaid & 36% had commercial insurance	68% not currently married	Not reported	36% English as second language. > 80% over the age of 55 yrs.
Ansari 2014	*N* = 17 Mean age 67; (range 43–84)	47	High cholesterol, hypertension, depression & smoking, COPD, arthritis	Range 1–7	Mostly Caucasian	Not reported, sample ranges from student to employed to retired, 11% unemployed and 1 person (5%) volunteer	65% live with spouse; 30% live alone; 5 (one person) live with a grandchild	9 had some high school education, the rest higher including 6 university	
Barstow 2015	*N* = 8. mean age was 79 years (range 66–92).	75	Arthritis- 88% hypertension- 62% hypotension- 25% cancer- 50% osteoporosis- 38% hearing impairment- 38% cardiac/circulatory system problems- 51% kidney problems- 37% digestive problems- 25% urinary system problems- 25% pulmonary problems- 13%	It was assessed using the General Health Questionnaire	White	Not reported	Not reported	Not reported	
Bayliss 2007	*N* = 16 Range: 31–70 years.	66	Osteoarthritis, depression & diabetes	38% had 4–9; 62% had 10–16 conditions	Ethnicity: 11% Hispanic/Latino; 88% other. Race: 90% white; 8% other; 2% black African American	76% less than USD 45,000; 17% more than 45,000.	53% married; 14% divorced/ separated; 2% never married; 29% widowed.	35% High-school educated; 10% some high school or less; 31% some college/ 2 year degree; 22% 4 year college degree.	
Bayliss 2003	*N* = 352 55% in age range of 65–74; 45% were 75 and above	81	Hypertension, COPD, chronic bronchitis, emphysema, asthma, musculoskeletal disorders, history of depression, vision problems, coronary arterial disease, migraine, obesity, gastroesophageal reflux, congestive heart failure, depression, osteoarthritis & diabetes, history of depression	Median: 4; range: 2–7	All white	7: < 15, 000; 8: > 15,000 ranging up to 60,000 USD	Not reported	At least high school education. High school graduate: 2; Some college: 7; College graduate: 5; Post-college: 2	
Beverly 2011	*N* = 32. mean 75.3 ± 7.4; range 60–88	56.3	Hypertension, retinopathy, hypercholesterolemia coronary artery disease neuropathy, cardiac arrhythmia, hypothyroidism, depression, myocardial infarction, asthma, chronic pain, presbycusis, stroke, chronic obstructive pulmonary disease, leukaemia, nephropathy, prostate cancer, insomnia, diabetes, arthritis and cancer	Mean 5.2; Range 3–9	100% white	Not reported, 94% retired	72% married	Mean 14.6 years of education; range 9–20	
Bunn 2017	*N* = 28, median age 82.5, range 59–94	36	Alzheimer’s disease 56%, mixed dementia 19%, vascular dementia 17%, Parkinson’s with dementia 8%, diabetes, stroke, vision impairment	Not reported	85% white-majority British white	Not reported	78% patients lived with a carer	Not reported	
Burton 2016	*N* = 30. Age 65–95	53	Diabetes, arthritis, cancer, stroke, hypertension, high cholesterol, angina, gout, cardiovascular disease	Not reported	All white except one South Asian	Not reported	15/30 live alone, 13 live with partner and 2 with a family member	Not reported	
Cheraghi-Sohi 2013	*N* = 30. mean 69; range 55–86	60	Osteoarthritis, cancer, diabetes	Mean 4; Range 2–9	Not reported	Not reported	Not reported	Not reported	
Clarke 2014	*N* = 35. Mean age of men 78.6. Mean women 80.3. Range 73–91	54	Back problems/ cataracts/ glaucoma/ heart disease, cancer, diabetes, arthritis	Range: 3–14; average 6	African 1, Asian/ South Asian 3, European 7, North American 23, South American 1	< 15,000 4, 15–24,999 4, 25–34,999 7, 35–44,999 3, 45–54,999 6, 55–64 1.65–74 1, 75–84 5	Participants lived in own home or retirement home. Currently married/ common law 13, divorced/ separated 5, never married 4, widowed 13	College/University 6, graduate school 7, high school 9, some high school 7, technical school 6	
Coventry 2014	*N* = 20 age Mean age 66.2 (54–88).	Not reported	Depression, COPD, cardiovascular disease, asthma, diabetes, arthritis	Median: 2.55; range: 2–4	Not reported	Not reported	Not reported	Not reported	
DiNapoli 2016	*N* = 28. Mean age 63.4 SD 6.3	22	Not identified on the excel	CIRG score mean 14.1 SD 3.3 &	64% White, rest not reported	4/28 working. 1 self-employed, 17 retired, 6 unemployed	61% non-married, 39% married	High-school educated (education mean: 13.71 ± 2.35)	
Fortin 2005	*N* = 25. mean 72.7 SD 8.2	60	Diabetes, others not reported	Mean 14.4 SD 4.5	Not reported	Only 20% had more than 50,000 income, the rest mostly between 10,000 and 40,000	48% were married, 32% widowed 8% divorced/ separated and 12% never married	76% had up to grade 12 education, 16% college and 4% university education	
Fried 2008	*N* = 66. Age 65 and older; > 75+ 6 participants	67	Hypertension, diabetes mellitus, ischemic heart disease, congestive heart failure, chronic lung disease, depression, arthritis, falls, urinary incontinence, osteoporosis	Median: 5; range 3–8;	76% white, 23% white, 1% other, 3% Latino	Not reported	48% lived alone. 39% married		
Gill 2014	*N* = 28. mean age: 82.3 (SD 7.7);	44	Not reported	Median 5 (SD 2.4)	Not reported	Not reported	82% of the caregivers were spousal caregivers	Most pts. and caregivers had more than high school education and lived in a single-family home	
Hansen 2015	*N* = 21. mean 77 (70–88)	47	Hypertension, lower back pain, diabetes, cancer, arthritis, osteoporosis, cardiac arrhythmias, cerebral ischemia, lower limb varicosities, prostatic hyperplasia, vision reduction, gout, intestinal diverticulosis, psoriasis, atherosclerosis, renal insufficiency, cardiac valve disorders, gallstones, cardiac insufficiency, anemias, neuropathies, migraine, urinary tract calculi, dizziness, hemorrhoids, gynecological problems	Not reported	Not reported	Not reported	Married: men 72%; women 40%; Widow: men 9%; women 40%; Divorced: men 9%; women 9%; Never married: men 9%	7 patients had a low education level; 7 had a medium level, and 6 had a high level	
Kuluski 2013	*N* = 28. mean age: 82.3 (7.7 SD)	44	Not reported	4.61 (SD 2.43)	96% Caucasian, 4% other	85% can support self financially	70% live in a single-family home; 15% apartment; 7% retirement home. 67% married; 33% other.	70% greater than high school; 30% high school diploma or less	
Lo 2016	*N* = 58; median age 67 (range 48–84)	29	Chronic kidney disease stage 3–5, depression, diabetes	Not reported	Majority of focus group participants were Caucasian (72.4%), South Asian (13.8%), Asian (10.3%), Pacific Islander (1.7%) or Hispanic (1.7%)	Not reported	Not reported	Not reported	
Loeb 2003	*N* = 37 older adults. Mean age 72; range 55–88	70	Not reported	Mean 4.5; Range 2–11	100% Caucasian	Not reported	100% independent and community dwelling.	Not reported	
Mason 2016	*N* = 37. Mean 76; range 55–92	38	Heart, respiratory, liver and renal failure, neurological conditions and mild dementia	Not reported	Not reported	Not reported	Not reported	Not reported	
McDonnall 2016	*N* = 131. Mean age 69.9 (range of 55 to 99)	62	Hearing and vision loss	Not reported	89% white, 4% African American, 3% American Indian, 1% Hispanic, 1.5% mixed and other unknown	Not reported	Private residence/ living alone 36%; private residence with spouse or others (53%); Retirement or assistive living facility (8%).	Not reported	
Morales-Asencio 2016	*N* = 18. mean age 73.6 years	61	Diabetes, arthritis, cancer, cardiovascular disease, chronic respiratory, congestive heart failure, COPD/ asthma/ renal impairment	Not reported	Not reported	Participants from working class neighborhoods, and in some cases, significantly limited living conditions	Two patients had no family caregiver. Participants lived in working class neighborhoods	Not reported	All were receiving home care
Naganathan 2016	*N* = 28. Mean 82.3	43	Not reported	Average 5; interquartile range of 3–7	Not reported	Not reported	70% lived in a single home.	70% had higher education	19% patients reported currently receiving home care; 96% receiving support from family caregiver, 70% from friends and neighbors, 26% from community programs
Noël 2005	*N* = 60. Age range 30s–80s no mean age provided	20	Not reported	Not reported	Majority white; African- Americans and Hispanics	Not reported	4 urban clinics and 4 rural clinics	Not reported	All veterans
Ravenscroft 2010	*N* = 20. 30% >74y; 45% 65-74y; 25% 45–64	55	Cardiovascular disease, chronic kidney disease, diabetes	Except one patient, all had 3 or more chronic conditions, majority with 3 or 4 stage chronic kidney disease	Caucasian 90%; black 10%	20% employed; 70% retired; 10% unemployed.	55% married; 30% widowed; 15% single. Participants: city - 85%; rural - 15%	60% beyond high school; 20% high school; 20%; less than high school	Home language - 80% English exclusively; 20% other
Richardson 2016	*N* = 33. 12% (51–60); 67% (61–70); 12% (71–80); 9% (81–90)	6	Diabetes, arthritis, cancer, hypertension, chronic pain, heart disease,	Mean = 6; Range 3–11	82% White; 18% black; 3% Hispanic; 97% non-Hispanic	12% under 10,000; 15% 10,000–19,999; 30% 20,000–39,999; 18% 40,000–49,999; 9% 50,000 + .	64% married; 33 divorced; 3 widowed	3% did not complete HS; 18% high school grad; 52% some college; 27% college or higher	
Roberge 2016	*N* = 10. 5 were 60 years or older.	50	Depression; anxiety; cardiovascular diseases; pulmonary conditions & musculoskeletal conditions	Not reported	Not reported	Not reported	7/10 married or lived with a partner	7/10 had high school degree or less	
Roberto 2005	*N* = 17. mean 76.1 (range 69–84)	100	Diabetes, heart disease, osteoporosis	mean 4.1; range 2–6	Not reported	Monthly income: two < 750; six 750–1000; one 1001–1299; Two 1300–1999; One 2000–2999; Two 3000–3900; three not reported.	11 Widowed; 1 Single; 4 Married; 1 Divorced. 11 lived alone, 1 with son, 1 with daughter, 4 with husband.	3 greater than high school; 12 high school; 2 < high school	
Ryan 2016	1805 were qualified as high need: 1274 Multiple complex chronic conditions; 379 under 65 disabled; 152 frail elderly. 82% 50 years and older with high needs.	52	Diabetes	Not reported	White, non-Hispanic: 64; Black, non-Hispanic: 10 & Hispanic: 15	The high needs population has lower level of income than the general US population	Not reported	The high needs population has lower level of education than the general US population	
Schoenberg 2011	*N* = 20. Mean age 55	85%	Heart disease or hypertension; arthritis; type 2 diabetes; cancer; stroke; and other illnesses	Mean of 4	95% white	65% less than $10,000 20% $10,001–15,000 5% $15,001–20,000 10% $20,001–25,000.	55% married. Most lived with at least one other person	15% had less than high school 25% attended some high school 55% earned a high school diploma or equivalent 5% had some postsecondary education	All unemployed. Average length of stay in county is 36 years. 70% had no health insurance. Those who did report insurance, indicated Medicaid, Medicare, or disability coverage.
Sheridan 2012	33 were aged 55–74 and 13 were 75+	50	Cardiovascular, COPD, congestive heart failure, depression, gout, diabetes, arthritis	Most had 3+ chronic conditions	32/42 were from ethic minority groups: 19 pacific, 12 Samoans, Maori 8 and Asian 3.	Lowest socioeconomic class quintile in Auckland	33 lived with family, 6 alone and 3 in residential care. 33 lived with family, 6 lived alone, 3 lived in residential care.	Not reported	
Zulman 2015	*N* = 53. Mean 59 (SD =11)	26	Diabetes, arthritis, cancer, hypertension, chronic pain, depression, headaches, PTSD, Lung/breathing problems, prostate problems	Mean 5 (SD 2)	White, non-Hispanic 81%; Black, non-Hispanic 6%; Hispanic 9%; Other, non-Hispanic 13%	<$50,000 43%; $50,001–$75,000 16%; >$75,001 41%	Not reported	High school degree or less 8%; some college + 44%; college degree or more 48%	
Health care professional characteristics									
Year and author	Sample size and mean age	Type of provider				Years of experience	Other		
Ancker 2015	*N* = 7, no age provided	2 Nurse Practitioners, 2 internists, 2 family medicine physicians; 1 emergency medicine physician				Not collected	4/7 Females		
Bardach 2012	*N* = 12, age range 31–47.	Family medicine, internal medicine and OB/GYN				3–22 years	They were all affiliated with a university health system but 5 practices in offsite community locations		
Barstow 2015	*N* = 59, no age provided	Occupational therapists				OT experience:< 10 year 25%, 10–20 45% & > 20 30%; Low vision experience: < 10 68.3%,10–20			
Bunn 2017	56 health care providers, no age provided	Family physicians; consultants with specialty in diabetes & VI; Rest not mentioned				Not reported			
Coventry 2014	*N* = 20, no age provided	16 family physicians and 4 Practice Nurses				18 (5–36)			
Gill 2014	*N* = 4, no age provided	4 family physicians from one family health team				Not reported			
Grundberg 2016	*N* = 25, age range 31–83.	Nurses. 2/25 were specialized nurses in mental health care. 4 had BScN degree, 6 MN degree. Most worked full time				range 4 months −34 years	2 were specialized RNs in mental health care. Most of them completed training in motivational interviewing		
Hansen 2015	*N* = 15, mean age 53.4 (range 39–65)	Family physicians				14.6 years (7–28)	Family physicians treated 500 to 749 patient every 3 months and 35.7% worked in single practices		
Kuluski 2013	*N* = 4, no age provided	Family physicians				Mean: 3; 3 practiced for at least 10 years, 1 physician had practiced 1 year.			
Naganathan 2016	*N* = 4, no age provided	4 family physicians				Not reported	46% physicians reported that patient currently receiving home care; 93% receiving support from family caregivers; 57% from friends and neighbours; 46% from community programmes.		
Roberge 2016	*N* = 18. Ae clinicians half were 30–39 years old	Clinicians (family physician, nurse, psychologist, social worker; *n* = 18)				56% had > 10 years’ experience	Clinicians felt at ease treating pts. with anxiety and depressive disorders. Sixteen had access to support of other mental health services and they had attended on average 1.7 days of continuing education related to mental health.		
Smith 2010		Family physicians				Not reported			
Caregiver characteristics									
Author and year	Sample size	Age	%female	Relationship to older adult		Education	Health	Other	
Bunn 2017	*N* = 33	Median age 65, range 46–90	82%	64% of carers were a spouse, 14% adult child		Not reported	Not reported	Carers: 85% white	
Gill 2014	*N* = 28	Mean age: 70.5; SD 11.3	79%	Spouse		Most had more than high school education	Not reported	Lived in a single-family home	
Kuluski 2013	*N* = 28	70.5 (SD 11.3)	82%	61% were spouses; 32% child; 3.5% sibling; 3.5% friend		Not reported	Not reported	Not reported	
Lo 2016	*N* = 8	No description provided except that they were carers of chronic kidney disease Stage 5 patients	Not reported	Not reported		Not reported	Not reported	Not reported	
Mason 2016	*N* = 17	Not reported	Not reported	Not reported		Not reported	Not reported	Not reported	
Morales-Asencio 2016	*N* = 18	Not reported	Not reported	spouse 72%, son/daughter 17%					
Naganathan 2016	*N* = 28	70.5 (SD = 11.3) years of age	82%	61% spousal caregivers		64% > high school		> 75% lived in a single family home	

*COPD* Chronic obstructive pulmonary disease

*OT* Occupational therapist

*PTSD* Post Traumatic Stress Disorder

*SD* Standard Deviation

MCC was determined mostly (24/32 studies) by health care providers, and/or staff assisting them, or the sample was drawn from clinical data bases and disease trajectories [26, 27, 30–32, 34–36, 38, 40–42, 44–50, 52, 53, 56, 59, 61]. Five studies did not explicitly state how MCC was established for the sample of patients during the recruitment process, although the inclusion criteria mentioned at least two or more conditions and the appropriate age range for older adults [33, 37, 51, 57, 58]. MCC was self- reported by patients in two studies [29, 43]. Frailty was not mentioned as a condition included in the MCCs.

The mean number of chronic conditions ranged from 2 to 4 in six studies [26, 34, 51, 52, 59, 61], from 5 to 7 in ten studies [30, 33, 37, 38, 41, 43, 46, 49, 55, 56], and 8 or more in one study [36]. Three studies reported range of number of chronic conditions [27, 29, 48]. The most reported conditions included hypertension, cardiovascular disease, chronic pain, osteoarthritis, COPD and cancer. Depression and other mental health conditions were also reported. Nineteen studies did not report the mean number of conditions [27–29, 31, 32, 35, 39, 40, 42, 44, 45, 47, 48, 50, 53, 54, 57, 58, 60].

Twenty-two studies reported the ethnicity of the study participants [26, 27, 29–33, 35, 37, 41–43, 47–49, 52, 53, 55, 57–60]. Most study participants were Caucasian or white, followed by ‘non-Hispanic’, Hispanic, black and South Asian populations. Other ethnicities were also reported in smaller proportions. Fourteen studies did not report ethnicity of the study participants [28, 34, 36, 38–40, 44–46, 50, 51, 54, 56, 61].

Nineteen of the 36 studies did not report the socio-economic status of the participants [28, 30–32, 34, 37–40, 42–44, 46, 47, 50, 54, 57, 60, 61]. Four studies included the employment status [27, 35, 48, 56], with most participants being unemployed. Income levels were reported from study participants belonging to varied socio-economic levels. Varied education levels were reported for study participants. Amongst all studies reporting education levels, most participants ranging from 35 to 70%, had high school education or higher [27, 29, 30, 33, 35, 36, 38, 41, 46, 48, 49, 51, 55, 56, 59], except for one study reporting education levels of high need adults being lower than the general population in the US [58]. Sixteen out of the 36 studies did not report education of the participants [26, 31, 32, 34, 37, 39, 42–45, 47, 53, 54, 57, 60, 61].

Living situation was reported with patients living with someone or alone. There was a wide variation in patients with MCC living alone, with a spouse and/or with family. Proportion of persons living with a spouse ranged from 13 to 82% [27, 30, 35, 36, 38, 40, 48–51, 59], while 30 to 50% of participants were living alone [27, 32, 37]. Urban-rural distribution was not reported by most studies, except one mentioning that there were 15% rural participants [48] and another mentioning that 50% of the clinics were rural [47]. No definitions of rurality were given.

The mean age of caregivers ranged from 69 to 71 [31, 38, 41, 46]. Three studies did not report the mean age of caregivers [42, 44, 45]. Eighty-two percent of the caregivers were female. Seventy-two percent were spouses of the older adults while 21% were adult children. In terms of HCPs, 1 study included nurse practitioners [26], physicians were included in 10 studies [26, 28, 31, 34, 38, 40, 41, 46, 50, 54], nurses were included in 4 studies [34, 39, 45, 50], psychologists were included in 1 study [50], pharmacists were included in 1 study [54], social workers in 1 study [50], and occupational therapists were included in 1 study [60]. The HCPs’ experience working with patients with MCC ranged from 4 months [39] to 36 years [34]. More detailed characteristics of the participants are presented in Table 2.

### Quality of the included studies

The quality assessment results are presented in Additional file 2: Table S2, available on line. The quality was moderate to good for most studies. We defined good quality as having a yes on all relevant quality criteria (*n* = 13), moderate as having items with no/can’t tell and the rest yes for relevant criteria (*n* = 23). There were no studies ranked as poor, which indicated a no on the majority of relevant quality assessment criteria. This rating scale allowed us to compare the different studies of different quality [62]. The rating scale allowed us to rate studies of different quality. Six studies used convenience samples [34, 39, 46, 56, 59, 60]. It was not always clear how the data were analyzed nor who analyzed the data [26, 29, 31, 61]. Also, it was not always clear how findings were influenced by researchers’ interactions with participants [31, 33, 35, 38, 44, 46, 48, 52, 54, 61].

### Thematic analysis of the findings

Five themes emerged from the data and there was convergence on most of the key themes between the older adults, the caregiver and the HCPs (Table 3). They included: 1) Need for information; 2) Need for coordination of services and supports; 3) Need for preventive, maintenance and restorative strategies, 4) Need for training to help manage the older adults’ complex conditions, and 5) Need for person-centred approaches. The few discrepancies within the themes will also be discussed.

**Table 3:** Overview of identified needs

First Author Publication Year	Actual Needs identified by older adults
Adeniji 2015	The needs which were identified most frequently (50% or higher) included: ‘Lack of information about my medical condition’ (55%) ‘Poor communication between different doctors or clinics’ (55%) ‘Lack of information about treatment options’ (60%) ‘Having to wait a long time to get an appointment for specialists (hospital doctors)’ (60%) ‘Lack of information about why my medication was prescribed to me’ (50%)
Ancker 2015	Some patients perceive medical records management as the team’s responsibility whereas other perceived it as their own. Patients make judgments about what data is relevant to their health. Managing transfers of medical information to solve problems such as health insurance denials is a tremendous amount of work that goes unrecognized.
Ansari 2014	New COPD diagnosis motivated participants to modify healthcare behaviors such as need to include physical activity and monitor diet; lack of communication between the participants and their physicians; expressed the need individualized plan and support for smoking cessation. The participants found managing MCC challenging due to the need to consume various medications and schedule various appointments, and voiced that after some time, the meds stop working. Participants who were most affected by arthritis and then developed COPD, found it quite challenging due it causing breathing difficulty, an additional problem with arthritis.
Barstow 2015	The patients describe their experiences but did not identify needs
Bayliss 2007	Self-reported health status: 12% excellent/very good; 38% good; 36% fair; 14% poor. Multivariable model was constructed: After adjusting for effects of multimorbidity, psychosocial factors were independently associated with health status and physical functioning. Greater disease burden, persistent depressive symptoms and financial constraints were associated with both lower health status and lower physical functioning. Symptoms and and/or treatments interfere with each other, and combined with a lower income level, were associated with lower physical functioning. Higher levels of patient-provider communication were associated with lower levels of physical functioning. Interactions were found between disease burden and communication, financial constraints, and the compound effect of conditions; additionally, impact of certain barriers may not be constant across the range of morbidity. Other factors that were significantly associated with the outcomes but did not contribute to the final models include: self-efficacy, being overwhelmed by a single condition; knowledge about medications and health literacy.
Bayliss 2003	patients were asked what barriers to their self-management was and the barriers included the self-care required for one condition could make the self-care for another condition difficult, the advice was sometimes incompatible, the symptoms influence each other and the medications can cause symptoms of the other disease worse, lack of knowledge, financial constraints to pay for all treatments, emotional stress of the diseases, need for adequate communication with providers, need for social support, need for understanding conditions and logistical issues dealing with multiple conditions.
Beverly 2011	Prioritizing health conditions: (i) Most patients acknowledge that complications of their diabetes motivated them to pay greater attention to their diabetes to diminish the progression of these complications. (ii) Patients reported prioritizing health conditions and severity or importance. (iii) Patients described feeling frustrated, confused, and overwhelmed in response to conflicting recommendations, particularly for diet, physical activity and medication regimens.
Bunn 2017	Both patients with dementia & caregivers expressed the need for continuity of care and involving them in the decision making process.
Burton 2016	In the interview asking the participant about their health. The participants who all had vision loss indicated challenges to accessing information, being dependent on family and friends to read letters and other information. The family physician was acting as another barrier to information and appointment attendance. Patients want their family physician to better coordinate care for persons with vision loss and other health conditions.
First Author Publication Year	Actual Needs identified by older adults
Cheraghi-Sohi 2013	Patient had a need for control and knowledge about their conditions. Patients had fluctuating priorities highlighting the importance of regular assessments during clinician-patient consultation to allow for better treatment planning. Patient priorities shift according to perceptions of control and/or interactions with clinical professionals. Focusing on management of only one single condition can lead to worse self-management.
Clarke 2014	They want their family physician to be thorough, they want to be referred to the expert, and they want their family physician to build a good trusting relationship for them. A third want their family physician to have a more person centered approach to decision making
Coventry 2014	Successful self-management in multimorbidity hinged on the interplay and interdependence between contextual factors related to1) patients capacity (access to resources), knowhow and confidence and physical and emotional abilities to accomplish self-management activities; 2) Responsibility was successful to self-management - patients had to be responsible for self-management tasks; 3) patients had to be motivated to manage their condition
DiNapoli 2016	Access to providers, asking for preference in provider, wanting their health care provider to build a doctor patient relationship, working together with the patient in a timely matter. To address mental health issues in the treatment for their chronic conditions. Advocate for the use of mental health services, advertise services available
Fortin 2005	Access to the family physician or specialist can be complicated due to automatic telephone messages, long waiting lings or the number of phone calls required. It creates anxiety. Also the waiting times in the ED are long and it is not clear when it is an emergency that they need to go to the ED (lack of capacity to determine the seriousness of the illness). Similarly there are long waiting times to see a specialist and the need for a referral is a barrier to access care. However, utilizing the family physician to determine whether ED or a specialist was needed could also speed up the access to care.
Fried 2008	1) Participants spoke about the concern with competing outcomes - the adverse effects of medications was a competing outcome that influenced their treatment decision making. 2) Participants spoke about global cross-disease outcomes (like preventing a stroke or heart attack) instead of disease specific outcomes (like lower blood pressure); Preference was for the treatment that would achieve the most desired outcome
Gill 2014	Patients reported lack of timely information and poor communication between health care providers and they had difficulty with symptom management and adhering to treatment recommendations. The patients complained about excessive wait times to see specialists. Furthermore, they had difficulty coordinating their care and medical trainees were even not consulting with their supervisor. Patients indicating not know how to prioritize their care and needs.
Hansen 2015	Patients expressed that there is no thorough explanations of the diagnoses by the specialists requiring them to go to their family physicians for clarity; need to have transfer of communication between family physicians and the specialists so family physicians are adequately informed of the patients’ MCC; difficulty understanding technical terms/jargons; patients expressed that they want to be seen by their family physicians as a person and not merely a number
Kuluski 2013	4 main themes: health maintenance; health improvement; behavior change; and preparation for future needs. ‘-Most patients wanted to prevent aggravating their health and chronic condition; these related to: avoiding inability to perform tasks because of pain; -Improvement matters to resume participating in physical and social activities that they were used to. -Behavior change was expressed as a need for losing weight and exercising, and being able to do more to relieve their caregivers; -Some expressed the need for preparing for the future which meant having home support, transitioning to a long term care facility. This was not always preferred; some wanted to stay and get help at home.
First Author Publication Year	Actual Needs identified by older adults
Lo 2016	Both patients and caregivers emphasized the key role of self-management, socio-economic situation and negative experiences as key in their health care as well as 5 health care service level factors empowerment of patient and the caregiver, access to care, poor coordination of care, continuity of care and poor recognition of psychological comorbidities. Being from a non-English speaking background led to difficulties in patient education, and self-management particularly with regard to nutrition. There is an extra financial burden due to due to transportation costs, paying for medication, marking and for maintaining a healthy lifestyle as well as community services that were used. The person who feels not well fatigue and disability impacted special life and relationships in a negative way. Psychiatric comorbidities such as depression make health self-management more difficult. Patients want more education to understand their disease, how to manage and the adverse outcomes. They appreciated support groups and sell-directed eLearning. The information should have been more combined for all diseases; the patient education material can be contradictory. There are problems with the coordination of care due to poor communication between hospital and primary care. Patients experience problems due to specialty boundaries, health care providers were unwilling to provide advice or offer help with problems that were not their scope. Patients experience a lack of continuity in care many different specialists with conflicting opinions. They felt one person should be in charge such as the PCP. Appointments should be scheduled so they don’t clash. Lack of access, lack of close by parking, too short consultation time, lack of interpreters, difficulty reaching health care providers,
Loeb 2003	Patients described periods of gaining, losing, and maintain capabilities through their experience of living with multiple chronic conditions. The main need was to maintain current capacity to perform activities of daily living. Following a period of declining capabilities (like a hospitalization); they worked towards a process of regaining capabilities to reestablish their previous health state. Coping strategies used to keep what they have included: relating with health care providers, medicating, exercising, changing dietary patterns, seeking information, relying on spirituality and/or religion, and engaging in life
Mason 2016	Complicated, confusing and sometimes unresponsive services. - Lack of care coordination and continuity among service providers - Attending clinics was physically demanding. - Frequent changes to medication changes cast doubts on their use. - Some perceived their care to be poorer because they are older (experiencing inequity). - Focused on living life to the fullest in the present. Thus, some participants avoided advance planning and only sought help when they were very ill or unable to cope. Deteriorating health was perceive as a manifestation of aging and thus delayed seeking help. Delaying services was furthermore seen as a way to preserve autonomy.
McDonall 2016	communication (understanding and being understood), transportation/mobility issues, access print, communication with health/service providers in the community, and training how to use technology, assistance with errands, information about assistive technologies for hearing, activities to participate in. In terms of the services they would have liked to have included transportation, older blind program, volunteers to assist with daily activities, and a senior center. They also discussed that health care providers should receive education on how to approach persons with a dual sensory impairment to maintain their dignity.
Morales-Asencio 2016	They had limited resources and there lots of barriers, lack of elevator in building, health care providers were not proactive in providing all information. Maintaining lifestyles was difficult. Implementing a treatment was difficult for some patients, and took time and effort. Lack of coordination of care, fragmentation of care. No clear care pathway when issues arise leading to ED visits. Not enough information given by provider for disease self-management. If support is offered it is helpful for adaption to the illness and treatment adherence. Health care services are fragmented and not adapted to persons with complex needs.
First Author Publication Year	Actual Needs identified by older adults
Naganathan 2016	Patients - Some felt a loss of independence and less in control - Patients emphasized wanting to remain at home and not be institutionalized- echoed by caregivers and physicians. - Sources of tension between patients, caregivers, and HCPs- discordance between patients’ perception of their independence and the amount of support are needed. Sometimes leading to caregiver burnout when family refuse help. - highlighted the importance of social networks.
Noel 2005	Illnesses had a significant impact on their daily life activities, work activities, social and family life. Uncertainty about their prognosis and inability to plan the future were important stressors. There were several problems with the health care system: 1) ling waits for referrals; 2) lack of continuity between clinics; 3) access to urgent care was not ideal; 4) poor communication with provider. Physicians had too many patients, were too busy or did not have enough support to provide care they needed. The time allowed for appointments was not long enough to discuss their health care needs. As they had many appointments scheduling was difficult to avoid impacting their work and family life. Patients felt specialists do not take their complaints seriously.
Ravenscroft 2010	-Fragmented care delivery: location of services across multiple locations, even within a single organization; lack of access to patient information leading to duplication of investigations, other problems; -Fragmentation complicated by health care provider’s time, information sharing with patients; logistical problems in keeping appointments such as transport, parking, etc. -MCC patients’ issues magnified with seemingly small health care issues, as these were repeated, increasing frustration levels, and finding solutions over and over again. Discovering the health system: -Process of ongoing discovery about the social structures within the health care system: patients perceived different parts, and constructed their own theories about it; providers difficult to differentiate between specialties, ranks and roles; regulation of interactions between them and providers; avoidance of MCC patients, referring them to others; reasonable expectations from the system were more often unrealistic; Managing the health care system: -patients strategized navigating the system; monitoring their care; they actively advocated through asking questions, voicing concerns and even ‘directing’ their providers; building and maintaining connections and relationships with trusted providers, and sought opportunities to end relationships with providers they did not trust; taking advantage of loopholes such as appointment cancellations to.
Richardson 2016	Veterans ranked their prioritization of their conditions according to: 1) perceived role of the condition in the body - that is, how the condition linked with the overall body function; 2) how the individual self-managed their conditions; 3) dealing with pain; 4) health care perception of which condition to prioritize Patients prioritized conditions by family history anticipating the same outcomes; impact on other conditions, daily activities such as mobility; and that have potential serious consequences if unmanaged. They also lacked knowledge about root causes of the conditions. Among self-management tasks, they prioritized conditions which required medical monitoring, felt in control of, activities based on financial costs, newer conditions requiring changes to daily routines. Patients prioritized pain management. Patients did not disclose their priorities to their HCPs. However, according to the patients, their HCPs have suggested which conditions to prioritize.
Roberge 2016	There were time constraints and patients hesitated to talk about their mental health. Not all patients wanted to talk about both their chronic condition and their mental health problem at the same family physician visit. Patients. felt stigmatized because of their mental health problem. Patients felts there are a lack of access to psychotherapy. Patients also reported lack of availability, costs, compatibility, language difficulty accessing services and their clinician’s lack of knowledge of available resources.
First Author Publication Year	Actual Needs identified by older adults
Roberto 2005	The women identified nine problems associated with their health concerns: pain, falls, functional limitations (e.g., activities of daily living [ADLs], instrumental activities of daily living [IADLs]), sleep disturbances, reduced energy, psychological distress (e.g., stress, worry), financial strain, medications, and compliance with treatment regime. The combination of problems attributed to different conditions increased the magnitude of the effect the women’s health had on their daily lives. Pain and a decline in energy frequently interfered with completion of daily activities. To compensate for this, many women reduced and slowed down the pace of activities they performed while emphasizing the importance of maintaining independence Appreciative of support from family members, at times the women received more help and advice than they preferred. Accepting health-related changes was not always easy for the women and often was complicated by the response and intended support of others
Ryan 2016	-Social isolation and unmet social needs: High needs patients showed emotional distress in last 2 years; 37% felt socially isolated, including lack of companionship, feeling left out, lonely and isolated as compared to 15% other adults in the sample. -Delaying care: 44% high needs patients reported delaying care due to an access issue- 22% transport as compared to 4% other adults; limited clinic hours; 29% due to inability to get appointments. -95% of high need patients had a regular doctor/clinic; 65% high need and 68% older adults were able to get answers to medical queries; -35% high need patients reported easily accessing care after hours without going to the emergency room, as compared to 53% other adults. --Assistance in managing conditions: -For stress, 43% could access counseling services when wanted; of the 53% high need patients needing multiple providers, 43% had a provider coordinating treatment; Of the 57% having issues with ADLs, 38% had someone to help them; 3/4th of which were relatives; -Insurance was also important: --Patient centered communication: 60% high-need patients had providers who fully engage in patient-centered communication, compared to other adults (52%). However, 82% of high-need adults were less likely to report that providers involve them in treatment decisions vs. 90% of others; 85% vs. 91% would listen carefully to them.
Schoenberg 2011	1) Participants viewed multimorbidity as more than the sum of its individual conditions. This led to worry over negative health consequences and conflicting and confusing treatment. 2) Community conditions including scarcity of personal resources, in adequate transportation to health care appointments, health care provider shortages, and insufficient healthy choices/resources undermined their self-management. 3) They managed their multimorbidity by settling into a routine that was often at odds with biomedical recommendations, but ones that worked for them.
Sheridan 2012	The visits with their family physician are short, mostly to describe pills, and lack of involved of practice nurses. Many reported feeling lonely, sad and suicidal. Most participants wanted to self-manage their conditions but they needed more information. The patients received conflicting messages from the different clinicians, feel that their provider do not communicate. Patients felt not being heard, there was difficulty in communicating and anger and mistrusts. Patients felt powerless.
Zulman 2015	3 Major themes 1. Managing a high volume of Information and Tasks: -High volume of records from multiple systems; absence of a comprehensive system in emergencies; Paperwork increases with each encounter with a provider; self-management routines to manage medicines, diets, etc.; -Health information: usually disease specific info available; condition interactions, risk of medication interactions, especially with multiple providers not available. Complicated medication regimes; patient may be the only person aware of it; multiple self-management tasks required throughout the day; multiple appointments to manage. -Communication: Complexity of MCC makes it difficult to seek care from new providers. 2. Coordinating multiple providers: almost no opportunity to involve multiple providers in a single discussion on management. 3. Serving as Expert and advocate: patients find themselves isolated/alone to resolve needs. -Peer support: difficult to find. -Caregivers: get overwhelmed with complexity and number of MCCs.
First Author Publication Year	Actual Needs identified by caregivers
Bunn 2017	Family members expressed the need to take charge to aid in getting continuity and access to services for their loved ones with dementia. They need to advocate for services and participate in medical decision making for the person living with dementia. They also played active role in coordinating care and services as well as navigate the healthcare system such as for arranging appointments and associated transportation needs, managing medications and serve as a means of communication between various providers. Caregivers reported formal support for persons living with dementia as inadequate. Lack of seamless sharing of information between various HCPs from different specialties. They identified a gap between the social care and healthcare and expressed the need for collaboration between them. As, this gap increases the risk for adverse events such as hypoglycemia.
Gill 2014	Caregivers also indicated long wait times, poor communication and lack of care coordination. It was difficult managing appointments with their work; they prefer to have a point person to talk to arrange care. Caregivers describe intentional noncompliance by the patient and due to complex city, facing stress from high risk decisions, feeling pressured and hopeless.
Kuluski 2013	6 themes, of which first 3 were the same as patient goals. For future needs preparation, they wanted the patients’ acceptance for services. -Health maintenance: keep up a social network and involved in activities, e.g. through regaining mobility and pain management; having a caregiver to rely on; acceptance of care from outside by the patient. -Doing tasks for the patient: some wanted to continue tasks for the patient, e.g. keeping appointments, medication management, nutrition; -Keeping the patient safe; with dignity so that patients don’t feel that they are being treated as invalid; which would also promote acceptance. Safety a major concern for dementia patients. -Helping patients maintain dignity, particularly at the end of life. -Stress management a major concern, to at least ‘keep sane.’
Mason 2016	Being a carer was not a choice. - carers experience physical and emotional stress
Naganathan 2013	Caregivers - Sources of tension about disagreement between patients and caregivers about future plans, and how to stay healthy and safe. - Emphasized the importance of formal supports for IADLS to alleviate caregiver burden and improve patient-caregiver relationships. - Felt immense burden with navigating healthcare system to obtain sufficient home care services.
First Author Publication Year	Actual Needs identified by health care providers
Ancker 2015	Providers need easy access to their patients’ information to make the best care decisions. Providers also talked about patient’s health literacy - for example patients selective reporting of information. Physicians often recognized that the patients understanding of the health care system influenced the way they shared their medical histories.
Bardach 2012	The physicians believed that their patients lacked the resources to follow prevention recommendations; the lack access to exercise, financial restraints to exercise or buy healthy food, lack of community resources, uninsured patients who have no access to resources. System barriers were also reported, time restraints, lack of reimbursement for preventative counseling. There is also a lack of care coordination particularly in the absence of EMR.
Barstow 2015	The HCPs described how comorbidities increased the number of visits, more visits cancelled and the need to collaborate with the caregivers well as the need for home visits. Nearly 60% identified a need for case coordination and many needs for referrals to other health care providers such as psychologists/counselor, physicians and diabetes educator
First Author Publication Year	Actual Needs identified by health care providers
Bunn 2017	HCPs used practices for alleviating the impact of living with dementia by reminding them of upcoming appointments, providing them with longer appointment times and same HCP that saw patient and carer. HCP need structured way of preparing for the progressing dementia and resultant worsening symptoms, which may lead to dropping out of the system leading to increased risk for adverse outcomes such as medication errors, caregiver burnout. HCPs spoke about the importance of personalizing care for the person living with dementia
Coventry 2014	Same needs as identified by patients because patients and HCPs data were analyzed together.
Gill 2014	The family physicians also discussed lack of access to care, poor communication and coordination, long wait times, and challenges with compliance, lack of home care for instrumental activities of daily living limitations, dealing with multiple specialists
Grundberg 2016	Patients often do not actively disclose mental health issues. There needs to be continuity of care and time to engage patients in dialogue about mental health. Common health issues in this population: depression, anxiety, sleeps problems and phobias. Patients need prompt psychiatric consultations. District nurses (DNs) need better teamwork with other HCPs so participants can increase their abilities in assessing and addressing mental health issues. DNs need to be more educated about mental health promotion activities and available resources for the patients. Older people with multimorbidity primarily lived alone and felt lonely which contributes to developing depression (especially affected women). Homebound seniors with few visitors are especially at risk for isolation and worsened mental health.
Hansen 2015	Specialists need to thoroughly inform family physicians about their patients’ diagnoses; due to lack of communication on diagnoses, family physicians spend a large sum of time to understand patients’ condition on their own and also to explain then to the patient; family physicians find this challenging due to a full waiting room; patients requires diseases to be explained at their level of understanding; patients identifies their issues based on symptoms and not necessarily according to prognosis e.g. vertigo
Kuluski 2013	Family physician goals ‘4 similar themes: -help maintain patient independence -heal, fix or improve symptoms when possible, -mobilize care for the patient and the caregiver -address safety issues. For the above goals, family physicians focused on preparing both patients and caregivers for worsening of health; maintaining independence; heal, fix or improve symptoms; particularly helping with acute exacerbations of conditions; family physicians emphasized supportive services and infrastructure, such as home care for safety, for both patients and caregivers; patient acceptance of these. For aging caregivers, stress was an important aspect to focus on to keep them healthy.
Naganthan 2013	Family physicians - physician reported a contradiction in that patients and caregivers refused additional support to stay at home when they desire to stay at home. - Caregivers who are heavily or exclusively relied upon by the patients tend to experience higher burden than those who receive support. - Some tension between physicians and families related to safety concerns. - Caregivers are viewed as key in navigating healthcare system and being the patients’ advocate
First Author Publication Year	Actual Needs identified by health care providers
Roberg 2016	The clinicians reported challenges with adherence as these patients required patient education and regular follow-up, they were often on a complex medication regime and they did not want more medication. Polypharmacy was also a challenge. The want more training on polypharmacy, more psychiatry rounds and more about different pharmacological options. The physicians reported it was difficult to obtain a consultation from a psychiatry in short term for patients when the pt. was on multiple meds and at risk of interactions but the condition was not deemed urgent. All physicians had difficulty communicating with private practice psychologists and that these psychologists could benefit from a better understanding of the nature and treatment of their pt.’s chronic diseases. The main barriers were the lack of mental health services, the delay accessing specialized services, less than optimal collaboration and communication between professionals, and training needs. For patients it included the burden of care (multiple treatments, frequent consultations) which influenced readiness to access additional services. The health and social service center had long waiting lists, complex pathways, many clinicians and often unspecialized services.
Smith 2010	5 main themes: 1. Multimorbidity and the link to Polypharmacy and ageing. • Multimorbidity a common phenomenon associated mostly with older age. Polypharmacy commonly associated with it, but not being given attention to, and which may add to multimorbidity. • Lack of distinction between multiple conditions and multiple risk factors was linked to the growth in preventive care; also clinical guidelines focused on single diseases which encouraged Polypharmacy. 2. Health system issues: -Lack of time for managing complex patients; increased workload; -Poor inter-professional communication, leading to fragmented care; between specialists, family physicians and pharmacists; latter felt isolated 3. Individual issues for clinicians: family physicians felt they were the coordinators of care; lacked clinical confidence dealing with complex issues; role of the practice nurse seemed unclear to them in managing MCC patients, since these were too complex for them to manage; making decisions in isolation from specialists; they regarded pharmacists having an important role, esp. for drug interactions; -Pharmacists wanted to be involved but felt overloaded; observed that family physicians don’t review medicines; decision making was at the specialist level, where hospital pharmacists could be involved; -Some suggested that specialists/hospitals were pushing their work on to primary care. -Inconsistencies: related to keeping patients under family physicians care, while at the same time wanting access to specialist care. -Clinical uncertainty related to stopping medications by both. -Family physicians felt inadequately managing MMC due to lack of time, and expertise. -Patient issues: Burden of MCC on care givers and patients was acknowledged; with the health system complicating care and patients becoming depressed; cognitive impairment was also an issue; depression and loneliness further burdened caregivers; while some patients took active interest in their care, particularly managing medications. -Potential solutions: Better models of care delivery, with more time for MCC; planning care better proactively; integrating rehabilitation programs; information sharing between providers; clear lines of responsibility.

*MCC* Multiple chronic conditions

### Need for information

The need for access to information was a theme that emerged the most often in the studies included in the review [26, 32, 38, 40, 45, 53, 56, 61]. Older persons with MCC spoke about the need for more information about their medical conditions. Specifically, they addressed the need for HCPs to use less technical terms and jargon [40], thorough explanations of diagnoses by specialists [40], comprehensive explanation of treatment options [40], and the rationale as to why certain medications were prescribed to them [56]. Patients believed that having a greater understanding of their conditions would help them better manage their conditions and gain greater control over their lives and be empowered [61]. However, many felt they did not have enough information for disease self-management [45]. Some older adults found that self-care required for one condition could make self-care for another condition difficult, as the advice was sometimes incompatible [29].

Patients also reported lack of timely information and poor communication between multiple HCPs [38], and often did not feel like they were being heard which led to distrust and feeling powerless [53]. Likewise, caregivers commented on the poor communication between HCPs and older adults, and because of the lack of seamless sharing of information between various team members and specialists, caregivers felt they needed to step in [31]. Because of the lack of information family members found themselves taking on an advocacy role, needing to participate in acquiring medical and service information as well as medical decision making [31].

HCPs also found accessing information about the older adult challenging [38]. Not being able to access the information made care decisions challenging [26]. They realized that the information patients shared with them was often related to their level of health literacy so selective reporting occurred [26]. HCPs also described issues related to access to information between fellow HCPs. Family physicians found that specialists did not thoroughly inform them about diagnoses, and hence family physicians spent a lot of time trying to understand the patient’s condition on their own and then having to explain it to the patient [40].

From some HCPs and older adults’ perspectives, older adults with dementia, mental health conditions and sensory impairments were found to be at higher risk for having needs not met [31, 32, 39]. Older adults with vision impairment indicated that they had additional challenges to accessing information and were dependent on family and friends to read information they received from their HCPs [32]. HCPs also spoke about older adults with mental health issues such as depression, anxiety and phobias experiencing difficulties accessing speciality services [39]. Moreover, HCPs perceived that older adults with dementia required more care such as reminding them about upcoming appointments and longer appointment times [31]. Caregivers for these patients also required more support as HCPs were concerned about caregiver burnout [31].

### Need for coordination of services and supports

Most of the participants reported the lack of coordination of services and supports significantly impacted the daily lives of older adults, caregivers and health care providers. The lack of coordination of services ranged from: lack of access to specialists [35, 36]; long wait times for referrals [56]; conflicting appointments [42]; short appointment time with family physicians [47]; referrals to different specialists with conflicting opinions [42]; duplication of investigations [48]; complicated and unresponsive services [44]; and no opportunity to involve multiple providers in a single discussion [55]. As a result, some patients experienced difficulty with symptom management and adhering to treatment recommendations [38]; and settled into a routine that was at odds with their primary physician’s recommendations [52]. Consequently, patients and caregivers became anxious as they were uncertain whether they were making the right choices [58], and they found themselves serving as the expert without the training [55], and felt isolated, frustrated and alone in resolving their issues [30, 58].

Caregivers mentioned that there was a lack of coordinated services in terms of formal support [31], home care services [46] and health maintenance activities [41] available for older adults. Caregivers expressed the preference for having a point person to arrange care [38] and provide continuity. For those caregivers still working, they experienced difficulties managing the care of their family member [38], but they felt they had no other option [31]. Some caregivers found themselves ensuring their family members kept their appointments, took over medication and nutrition management, and were instrumental in ensuring the person maintained their dignity, particularly at the end of life [41]. Taking on this coordinator role was a source of tension between some older adults and their caregivers as they had conflicting ideas about future plans, and how to stay healthy and safe [46].

HCPs [38, 50, 60] also identified the need for better coordinated services and supports and recommended a case coordinator [60]. Physicians identified that older adults with MCC needed to be seen by other specialists such as psychologists, diabetes educators, or mental health specialists and a coordinator could assist with this task [60] because there were often complex pathways to negotiate in order to be seen by these specialists [50]. Also, some family physicians found coordinating with multiple specialists challenging [38]. Fragmented care between specialists, GPs and pharmacists left pharmacists feeling isolated” (Smith et al. 2010).

As found in many of the articles in the review, the lack of coordination of services led to stress for older adults, caregivers and HCPs [31, 38, 44, 46, 48, 55]. Caregivers described that they had experienced their family members being non-compliant because of the complexity they were facing and the high-risk decisions they felt they had to make, which caused them to feel pressured and hopeless [38]. Because systems were so fragmented, many persons with MCC had to repeat their issues to different providers and with this came increasing levels of frustration, as they sought solutions over and over again [48]. Caregivers also became overwhelmed with the complexity and number of chronic conditions their family member had [55], or if their symptoms worsened [31], and they felt burdened by navigating the health care system to obtain services [46], all of which eventually could lead to burnout especially if the family member of older adults refused the help suggested [46]. As Mason [44] pointed out, being a caregiver was not a choice, and they often experienced physical and emotional stress. Bunn and colleagues (2017) noted a discrepancy between HCPs attempts to coordinate care for older adults with dementia and caregivers’ perceptions of these efforts. HCPs perceived they took extra time for older adults and their caregivers, reminding them about their appointments, and preparing for worsening dementia related issues, whilst family members felt more time was required with their HCPs, better coordination was needed as they felt they had to navigate care such as arranging appointments and sharing of information with different HCPs.

### Need for preventive, maintenance and restorative strategies

The need to prevent further deterioration in daily living, maintain current levels of function and restore any lost abilities was articulated by older adults, and echoed by caregivers and HCPs in a few of the studies [27, 41, 45, 46, 49]. Most older adults wanted to prevent the aggravation of their health and chronic conditions [41]. They also articulated the need to maintain current capacity to perform activities of daily living [43] but for many, maintaining lifestyles was difficult [45]. A new diagnosis often motivated older adults to modify their health care behaviors and daily routines [49] such as including physical activity into their daily regime and monitoring their diet [27], however this was difficult for some older adults, and took time and effort [45]. In order to accomplish this modification, a behavior change was often required and if support was offered it was helpful for adoption of the new treatment [45]. For some caregivers, their role included motivating their family member to make these changes, but they struggled with how to do this well and it caused a source of tension between the older adult and their caregiver [46]. Naganathan and colleagues (2016) highlighted discrepancies between caregivers and older adults’ future plans on how to stay healthy and remain at home. There was disagreement on how each participant group perceived their independence and how much support was required to live at home. Family physicians were also not sure on how to handle these differences in perceptions as patients and caregivers often refused additional support which raised concerns about remaining safely at home without caregiver burnout resulting as an outcome.

Physicians in a few of the studies knew that their patients lacked the resources to follow prevention recommendations [28]. There was a recognition by HCPs that for some older adults their inability to buy healthy foods, financial restraints to pay for treatments [29], lack of community resources, or being uninsured held many back from participating in prevention [28]. Additionally, physicians also did not receive reimbursement for preventive counselling [28] nor for the longer appointment times required. Family physicians thus aimed to provide guidance by helping with acute exacerbations of conditions and improving symptoms [41]. In addition, they felt their role included emphasizing supportive services, such as home safety so the older adults could maintain function as long as they could, and to prepare both patients and caregivers for worsening of health when the time would come [41]. However, some physicians found some support services lacking such as home care which was essential to maintaining function of older adults [38].

### Need for training to help manage the older adults’ complex conditions

A need identified by all three groups was for training to help manage older adults’ complex conditions and to plan for the future. Older adults wanted more education to understand their disease, how to manage adverse outcomes and when offered, they appreciated support groups and self-directed eLearning [42]. They also wanted training on how to use technology [57]. Older adults also wanted HCPs to receive education on how to approach persons with dual sensory impairment to maintain their dignity [57]. Both older adults and caregivers, perceived a need for more education and training on health literacy and their medications [59].

Health care providers commented that polypharmacy was a challenge and that they wanted more training on different pharmacological options [50]. In addition, some physicians felt they lacked clinical confidence dealing with multiple complex issues, as clinical guidelines focused on one single condition leading to polypharmacy [54].

### Need for person-centred approaches

The need for more person-centred approaches to service delivery was highlighted in many of the reviewed articles by older adults and their caregivers [31, 33, 40, 48, 53]. Older adults wanted to be seen as a person and not merely as a number [40]. They highlighted the need for patient-centred communication and to be involved in treatment decisions and feel listened to during their interactions with HCPs. Clarke [33] highlighted that the older adults wanted the primary care provider to build a good trusting relationship with them and to have a more person-centred approach to decision making. Older adults wanted to build and maintain connections and relationships with trusted providers and sought to end relationships with providers they did not trust [48]. Many persons felt that they were not being heard, which led to distrust [53] in the relationship and feeling powerless. In only one study reviewed, HCPs did suggest that personalizing care for persons living with dementia was essential [31] and they made sure to see both the patient and caregiver at each appointment. For those physicians who took a person-centred approach, such as, by taking time and listening to patients, there were repercussions, as often a full waiting room of patients were left in the clinic waiting for their appointments [40].

### Structural and social determinants of health

Structural and social determinants were examined to identify how they influenced needs of older adults (see Table 4). Of the social and structural determinants of health included in the selected studies, twelve mentioned education which is often a proxy for health literacy concerns [26, 27, 38, 43–45, 47, 50–53, 56, 58], nineteen mentioned access issues [27–29, 32, 35, 36, 38, 40, 41, 44–47, 50, 52, 54, 56–58], and fifteen highlighted the impact of socioeconomic status on needs [27–30, 34, 35, 42, 43, 49–53, 58, 59].

**Table 4:** Overview of Social and structural determinants of Health impacting health and social care needs in older adults with multiple chronic conditions

Study author and year	SES	Gender	Education	Ethnicity	Living circumstances (rural /urban)	Living situation (alone or not)	Social Support/ network	Access issues
Adeniji 2015			X					X
Ancker 2015		X	X					
Ansari 2014	X	X	X			X		X
Bardach 2012	X							X
Barstow 2015								
Bayliss 2008	X							
Bayliss 2003	X							X
Beverly 2011	X			X				
Bunn 2017						X		
Burton 2016								X
Cheraghi-Sohi 2013						X	X	
Clarke 2014		X						
Coventry 2014	X						X	
DiNapoli 2016	X	x				X		X
Fortin 2005								X
Fried 2008								
Gill 2014			X				X	X
Grundberg 2016		X				X		
Hansen 2015								X
Kuluski 2013							X	X
Lo 2016	X			X	X	X		
Loeb 2003	X		X			X	X	
Mason 2016			X			X		X
McDonnall 2016								X
Morales-Asencio 2016			X					X
Naganathan 2016								X
Noël 2005			X					X
Ravenscroft 2010				x				
Richardson 2016	X							
Roberge 2016	X		X	X	X			X
Roberto 2005	X		X				X	
Ryan 2016	X	X	X	X			X	X
Schoenberg 2011	X	X			X		X	X
Sheridan 2012	X		X	X			X	
Smith 2010								X
Zulman 2015								

In contrast, only three studies cited the link between living circumstances and health and social needs [42, 50, 52]. Some studies mentioned gender [26, 27, 33, 35, 39, 52, 58], ethnicity [30, 42, 48, 50, 53, 58], living situation [27, 31, 35, 39, 42–44, 61] and having a social support network [34, 38, 41, 43, 51–53, 58, 61] in relation to the health and social care needs for older adults with MCC. In summary, it would appear that socioeconomic status, education and access to the health care system were the predominant structural and social determinants of health that influenced the needs of older adults with MCC. Gender, ethnicity, living situation and social support received less attention.

### Stakeholder consultation

All attendees agreed that the identified themes resonated with their experiences. A concern was expressed that few of the studies included older persons over the age of 85 years and emphasized they may have very different needs as they may likely be housebound. Stakeholders pointed out that there needs to be a discussion with patients and caregivers on goals of care in the final years and advanced care planning factoring in advanced age, number of disease conditions, level of function and frailty. They all agreed that access to services and supports were a concern and expressed that older persons with MCC are treated differently in the healthcare system, for example not gaining access to rehabilitation to restore function, which may be suggestive of ageism. Stakeholders felt that the needs of older adults with MCC are much more complex today as compared to several years ago but the level of staff expertise available to them has not kept pace, as care is often provided by unregulated professionals, especially in the community. Stakeholders agreed that caregivers have unique needs and there is little regard to their capacity and remuneration for the work, leading to caregiver stress. Finally, one older adult with MCC strongly advocated that we stop applying band-aid solutions (i.e., improving communication between health care providers and older adults) and instead, focus on re-inventing how care is organized and delivered.

## Discussion

Our scoping review highlighted that, of the 36 studies reviewed, there was convergence between needs of older adults from the perspectives of older adults, caregivers and HCPs. The findings from our review revealed that there is a need for access to information, coordination of services and support, strategies for prevention, maintenance and restoration, training and a focus on person-centred approaches. Our findings also suggest that older adults wish to be seen as a person and not merely a collection of disease conditions. Lack of coordination and access to information was prominently highlighted in the studies as well as the stakeholder consultation. The occurrence across various countries and jurisdictions suggests that fragmented services is a prevalent issues warranting further attention.

Specific to structural and social determinants of health, socioeconomic status was one of the main concerns and it was related to older adults’ ability to pay for treatments [59], and the extra financial burden that MCC had on the costs of transportation, medication, and maintaining a healthy lifestyle. Access to services was also a major determinant and therefore, coordinating services within and across sectors and considering the needs of all individuals is essential to optimize care. Educational level and health literacy were also highlighted by HCPs as a barrier to effective management of MCC [55]. Gender and ethnicity were also cited, as non-English speaking backgrounds also led to difficulties in patient education and self -management [42]. Similarly, in a recent review by Northwood [10], gender, education, and the health system were found to be most commonly cited determinants of social determinants of health that impact persons with MCC. Less commonly cited were living situation, however, living alone and being homebound with MCC were also seen as contributing to developing depression, especially in women [39]. Furthermore, social isolation was a concern and Ryan [58] found that there was a relationship between social isolation and multiple unmet needs for older adults with MCC. Finally, living in rural areas may result in scarcity of personal resources, lack of family support, inadequate transportation, health care provider and service shortages, and insufficient healthy food choices and resources which could undermine management of MCC in community dwelling older adults.

To a certain degree the findings from this review are in line with priorities for improving care set out in the WHO framework for integrated, people centred health services [63]. This review adds specific details about how needs can be met including service coordination, making sure information goes from provider to provider, continuity, improved access, and assistance navigating the system. In sum, what is required is a restructuring of the health and social care system to incorporate an integrated care approach. This type of approach would result in a HCP responsible for the care coordination of a care plan that has been developed with the older adult and their caregiver to address their priorities and thus, would be more person-centred and tailored to their needs, goals and priorities. In addition, it would involve the interprofessional team across sectors that share decision-making and communicate to implement the integrated care plan, coordinating the services from different providers and thus reducing the conflicting advice from multiple providers.

Empowering patients and families to self-manage is an important aspect of the care delivery. Promising integrated care models are currently being tested such as the IMPACT clinic [64] and the Guided Care Model [65]. Stakeholder consultation also suggested the presence of discrimination and social injustice due to advanced age. Incorporating patient-centred outcome measures can strengthen governance and accountability to increase the quality of healthcare for older adults with MCC. Finally, there requires a move from hospital-based and curative care to outpatient and preventive care e.g. establishing interprofessional teams and empowering primary care teams through allocating increased healthcare funding to be able to optimize their resources.

In terms of future direction for research, most of the views of the needs of older adults with MCC were consistent with those of their care giver and HCPs and there were few areas of divergence. Practice and research in the future could focus on ensuring the views of older adults and their caregivers are noted by HCPs as this discrepancy most likely influences outcomes. The study by Naganathan et al. (2016) highlighted a key discrepancy about safety concerns at home and supports required to age at home successfully. More research is required to focus on the dignity of risk and how to provide supports to older adults in their homes that are meaningful to them, and may include more social support interventions such as friendly visitor programs versus a focus only on health care needs. In addition, most of the studies in this review were qualitative in nature and thus no relationships between older adults’ needs with MCC and outcomes were found, nor predictors of these needs. Finally despite focusing on health and social care needs of older adults, few social care needs were identified. This gap points to a promising area for future research.

The strengths of the scoping review include a comprehensive search of electronic data bases carried out by expert health sciences librarians, two reviewers for data abstractions and multiple checks of the source articles. Given the large number and range of older adults with varying types of multiple chronic conditions included in our selected studies, our findings are fairly representative of persons living with MCC. In addition, the convergence of our findings from the three perspectives which resonated with the stakeholder group helps to validate the results. Due to the number and heterogeneity of the studies retrieved, decisions were made to focus on only the ‘needs’ of older adults and not their preferences or lived experience.

## Conclusion

Consensus was found among the three perspectives in terms of needs of older adults with MCC. Older adults have needs at the individual, home, and system levels. Issues related to access for information and adequate support and services are pervasive for persons with MCC. Structural and social determinants of health are important to consider when addressing needs and solutions for older adults. Future studies should include developing and testing integrated models of care, and determine if access, information and person-centered approaches utilizing intersectoral strategies can be realized.

## Additional files

Additional file 1: **Table S1.** Medline Search. (DOCX 38 kb)

Additional file 2: **Table S2.** Quality Assessment using the Mixed Methods Appraisal Tool (MMAT) *. (DOCX 33 kb)

## Abbreviations

CI: Cognitive Impairment
HCPs: Health Care Providers
MCC: Multiple Chronic Conditions
MMAT: Mixed Methods Appraisal Tool
SES: Socioeconomic Status
WHO: World Health Organization
