# Supplementary material for: Identifying and understanding the health and social care needs of older adults with multiple chronic conditions and their caregivers: a scoping review
Source: BMC Geriatr. 2018 Oct 1;18:231. doi: 10.1186/s12877-018-0925-x (PMC6167839; doi:10.1186/s12877-018-0925-x)
Supplement: Supplementary file 1 — Table S1. Medline Search. (DOCX 38 kb) [file 12877_2018_925_MOESM1_ESM.docx]

# **Table S1: Medline Search**

Database: **Ovid MEDLINE: Epub Ahead of Print, In-Process & Other Non-Indexed Citations, Ovid MEDLINE® Daily and Ovid MEDLINE®**1946-Present

Search Strategy:

| **#** | **Searches** | **Results** |
| --- | --- | --- |
| 1 | Comorbidity/ | 88841 |
| 2 | MCC.tw,kf. | 4350 |
| 3 | polypharmacy.tw,kf. | 5089 |
| 4 | multidiseas*.tw,kf. | 38 |
| 5 | (polymorbid* or polydiseas* or polypath* or pluralpatholog*).tw,kf. | 438 |
| 6 | (dual adj3 diagnos*).tw,kf. | 2107 |
| 7 | (multi-morbid* or multimorbid*).tw,kf. | 3106 |
| 8 | ((multiple or concomitant or simultaneous or concurrent or co-occur* or co-exist* or coexist* or (two adj1 more)) adj2 (morbid* or comorbid* or chronic*)).ti,kf. or ((multiple or concomitant or simultaneous or concurrent or co-occur* or co-exist* or coexist* or (two adj1 more)) adj1 (chronic* or morbid* or comborbid*)).ab. | 4179 |
| 9 | Vulnerable Populations/ or Multiple Chronic Conditions/ | 8005 |
| 10 | Population Groups/eh [Ethnology] | 579 |
| 11 | exp Chronic Disease/eh [Ethnology] | 374 |
| 12 | american native continental ancestry group/ or alaska natives/ or indians, north american/ or inuits/ | 16840 |
| 13 | (aborigin* or maori* or indian* or inuit* or inuk* or indigenous or native* or eskimo* or metis).tw,kf. | 283285 |
| 14 | (native* adj (alask* or american* or canadian* or hawaii)).tw,kf. | 4574 |
| 15 | (pacific adj islander*).tw,kf. | 2921 |
| 16 | (american adj (indian* or samoan*)).tw,kf. | 5798 |
| 17 | (first adj (nations or nation)).tw,kf. | 1337 |
| 18 | (torres adj strait adj islander*).tw,kf. | 1060 |
| 19 | Oceanic Ancestry Group/ | 8716 |
| 20 | tribe*.tw,kf. | 7619 |
| 21 | "Delivery of Health Care"/ | 77153 |
| 22 | Health Priorities/ | 10169 |
| 23 | "health services needs and demand"/ | 50005 |
| 24 | Patient Preference/ | 5488 |
| 25 | patient reported outcome measures/ | 336 |
| 26 | "surveys and questionnaires"/ | 381988 |
| 27 | "quality of health care"/ or "outcome and process assessment (health care)"/ | 89246 |
| 28 | Health Equity/ | 227 |
| 29 | Healthcare Disparities/ | 11578 |
| 30 | ((health* or social*) adj2 (plan* or priorit* or need or needs or research)).ab. | 71855 |
| 31 | ((health* or social*) adj4 (plan* or priorit* or need or needs or research)).ti,kf. | 34126 |
| 32 | ((setting* or patient*) adj2 (need or needs or prefer* or priority or priorities or plan or planning)).ab. | 48957 |
| 33 | ((setting* or patient*) adj4 (need or needs or prefer* or priority or priorities or plan or planning)).ti,kf. | 11549 |
| 34 | (lived adj5 experience*).ti,kf. | 1445 |
| 35 | (lived adj2 experience*).ab. | 3666 |
| 36 | (outcome* adj4 improv*).ti,kf. | 11397 |
| 37 | (outcome* adj2 improv*).ab. | 84127 |
| 38 | Geriatrics/ or Veterans/ | 42000 |
| 39 | adult.mp. or middle aged.sh. or aged.sh. or (middle adj age*).tw,kf. | 6825587 |
| 40 | adult/ or exp aged/ or middle aged/ | 6367293 |
| 41 | Health Services for the Aged/ | 16755 |
| 42 | Health Services, Indigenous/ | 2692 |
| 43 | exp Animals/ not (Humans/ and exp Animals/) | 4397724 |
| 44 | or/21-37 [Health priorities] | 805136 |
| 45 | or/38-40 [Adults] | 6874331 |
| 46 | (44 and 45) or 41 [String 1] | 394287 |
| 47 | or/1-20 [MCC] | 409790 |
| 48 | (47 and 44) or 42 [String 2] | 28033 |
| 49 | 45 and 48 [String 2 AND Adults – String 3] | 16647 |
| 50 | 46 and 49 [String 3 AND String 1] | 16228 |
| 51 | limit 50 to yr="2000 - 2017" | 15009 |
| 52 | 51 not 43 | 15000 |
| 53 | **limit 52 to (dutch or english or french or german)** | **14589** |
