# Supplementary material for: Identifying and understanding the health and social care needs of older adults with multiple chronic conditions and their caregivers: a scoping review
Source: BMC Geriatr. 2018 Oct 1;18:231. doi: 10.1186/s12877-018-0925-x (PMC6167839; doi:10.1186/s12877-018-0925-x)
Supplement: Supplementary file 2 — Table S2. Quality Assessment using the Mixed Methods Appraisal Tool (MMAT) *. (DOCX 33 kb) [file 12877_2018_925_MOESM2_ESM.docx]

**Table S2 Quality Assessment using the Mixed Methods Appraisal Tool (MMAT) ***

| MMAT item& | Clear research question? | Data appropriate for research question? | Qualitative | | | | Quantitative Descriptive | | | | Mixed methods | | |
| --- | --- | --- | --- | --- | --- | --- | --- | --- | --- | --- | --- | --- | --- |
| Author, publication year (reference) |  |  | 1. Data relevant to research question? | 2. Data analysis appropriate? | 3. Context of data and findings | 4. Researchers’ interaction with participants | 1. Sampling Strategy | 2. Sample representativeness | 3. Measurement | 4. Response rate (>60%) | 1. Sampling Strategy relevant | Integration relevant? | Consideration to the limitations associated with the integration of data/ results? |
| Adeniji 2015 | Yes | Yes |  |  |  |  | Yes | Yes | Yes | No (33%) |  |  |  |
| Ancker 2015 | Yes | Yes | Yes | Can’t tell | Yes | yes |  |  |  |  |  |  |  |
| Ansari 2014 | Yes | Yes | Yes | Yes | Yes | Yes |  |  |  |  |  |  |  |
| Bardach 2012 | Yes | Yes | Yes | Yes | Yes | Yes |  |  |  |  |  |  |  |
| Barstow 2015 | Yes | Yes | Yes | Yes | Yes | Yes | Yes | Yes | Yes | No (52%) | Yes | Yes | Yes |
| Bayliss 2007 | Yes | Yes |  |  |  |  | Yes | Yes | Yes | No (47%) |  |  |  |
| Bayliss 2003 | Yes | Yes | Yes | Can’t tell | Can’t tell | Yes |  |  |  |  |  |  |  |
| Beverly 2011 | Yes | Yes | Yes | Yes | Yes | yes |  |  |  |  |  |  |  |
| Bunn 2017 | Yes | Yes | Yes | No | Yes | Can’t tell |  |  |  |  |  |  |  |
| Burton 2016 | Yes | Yes | Can’t tell | Yes | Yes | Yes |  |  |  |  |  |  |  |
| Cheraghi-Sohi 2013 | Yes | No | Yes | No | Yes | Can’t tell |  |  |  |  |  |  |  |
| Clarke 2014 | Yes | Yes | Yes | Yes | Can’t tell | Can’t tell |  |  |  |  |  |  |  |
| Coventry 2014 | Yes | Yes | Yes | Yes | No | Yes |  |  |  |  |  |  |  |
| DiNapoli 2016 | Yes | Yes | Yes | Yes | Yes | Can’t tell |  |  |  |  |  |  |  |
| Fortin 2005 | Yes | Yes | Yes | Yes | Yes | Yes |  |  |  |  |  |  |  |
| Fried 2008 | Yes | Yes | Yes | Yes | Yes | Yes |  |  |  |  |  |  |  |
| Gill 2014 | Yes | Yes | Yes | Yes | Yes | No |  |  |  |  |  |  |  |
| Grundberg 2016 | Yes | Yes | Yes | Yes | Yes | Yes |  |  |  |  |  |  |  |
| Hansen 2015 | Yes | Yes | Yes | Yes | No | Yes |  |  |  |  |  |  |  |
| Kuluski 2013 | Yes | Yes | Yes | Yes | No | Yes |  |  |  |  |  |  |  |
| Lo 2016 | Yes | Yes | Yes | Yes | Yes | Yes |  |  |  |  |  |  |  |
| Loeb 2003 | Yes | Yes | Yes | Yes | Yes | yes |  |  |  |  |  |  |  |
| Mason 2016 | Yes | Yes | Yes | Yes | Yes | Can’t tell |  |  |  |  |  |  |  |
| McDonnall 2016 | Yes | Yes |  |  |  |  | Yes | Yes | Yes | Not reported |  |  |  |
| Morales-Asencio 2016 | Yes | Yes | Yes | Yes | Yes | Yes |  |  |  |  |  |  |  |
| Naganathan 2016 | Yes | Yes | Yes | Yes | No | No |  |  |  |  |  |  |  |
| Noël 2005 | Yes | Yes | Yes | Yes | Yes | Yes |  |  |  |  |  |  |  |
| Ravenscroft 2010 | Yes | Yes | Yes | Yes | Yes | Can’t tell, missing |  |  |  |  |  |  |  |
| Richardson 2016 | Yes | Yes | Yes | Yes | Yes | Yes |  |  |  |  |  |  |  |
| Roberge 2016 | Yes | Yes | Yes | Yes | Yes | yes |  |  |  |  |  |  |  |
| Roberto 2005 | Yes | Yes | Yes | Yes | Yes | Yes |  |  |  |  |  |  |  |
| Ryan 2016 | Yes | Yes |  |  |  |  | Yes | Yes | Can’t tell | Can’t tell |  |  |  |
| Schoenberg 2011 | Yes | Yes | Yes | Yes | Yes | No |  |  |  |  |  |  |  |
| Sheridan 2012 | Yes | Yes | Yes | Yes | Yes | Yes |  |  |  |  |  |  |  |
| Smith 2010 | Yes | Yes | Yes | Yes | Yes | No |  |  |  |  |  |  |  |
| Zulman 2015 | Yes | Yes | Yes | Yes | Yes | Yes |  |  |  |  |  |  |  |

*As there was no study that had a quantitative randomized controlled trial or quantitative non-randomized controlled trial design, the quality criteria of the MMAT for those 2 study designs were omitted from the table
